# Benchmark dose analyses of multiple genetic toxicity endpoints permit robust, cross-tissue comparisons of MutaMouse responses to orally delivered benzo[a]pyrene

**DOI:** 10.1007/s00204-017-2099-2

**Published:** 2017-11-24

**Authors:** Alexandra S. Long, John W. Wills, Dorothy Krolak, Matthew Guo, Stephen D. Dertinger, Volker M. Arlt, Paul A. White

**Affiliations:** 1 Mechanistic Studies Division, Environmental Health Science and Research Bureau, Health Canada, 50 Colombine Driveway, Tunney’s Pasture, A/L 0803A, Ottawa, ON K1A 0K9 Canada; 2 Department of Biology, University of Ottawa, Ottawa, ON Canada; 3 Litron Laboratories, Rochester, NY USA; 4 Analytical and Environmental Sciences Division, MRC-PHE Centre for Environment and Health, King’s College London, London, UK

**Keywords:** Benzo[a]pyrene, Mutation, Genotoxicity, Clastogenicity, DNA adducts, BMD

## Abstract

Genetic damage is a key event in tumorigenesis, and chemically induced genotoxic effects are a human health concern. Although genetic toxicity data have historically been interpreted using a qualitative screen-and-bin approach, there is increasing interest in quantitative analysis of genetic toxicity dose–response data. We demonstrate an emerging use of the benchmark dose (BMD)-approach for empirically ranking cross-tissue sensitivity. Using a model environmental carcinogen, we quantitatively examined responses for four genetic damage endpoints over an extended dose range, and conducted cross-tissue sensitivity rankings using BMD_100_ values and their 90% confidence intervals (CIs). MutaMouse specimens were orally exposed to 11 doses of benzo[a]pyrene. DNA adduct frequency and *lacZ* mutant frequency (MF) were measured in up to 8 tissues, and *Pig*-*a* MF and micronuclei (MN) were assessed in immature (RETs) and mature red blood cells (RBCs). The cross-tissue BMD pattern for *lacZ* MF is similar to that observed for DNA adducts, and is consistent with an oral route-of-exposure and differences in tissue-specific metabolism and proliferation. The *lacZ* MF BMDs were significantly correlated with the tissue-matched adduct BMDs, demonstrating a consistent adduct conversion rate across tissues. The BMD CIs, for both the *Pig*-*a* and the MN endpoints, overlapped for RETs and RBCs, suggesting comparable utility of both cell populations for protracted exposures. Examination of endpoint-specific response maxima illustrates the difficulty of comparing BMD values for a fixed benchmark response across endpoints. Overall, the BMD-approach permitted robust comparisons of responses across tissues/endpoints, which is valuable to our mechanistic understanding of how benzo[a]pyrene induces genetic damage.

**Electronic supplementary material:**

The online version of this article (doi:10.1007/s00204-017-2099-2) contains supplementary material, which is available to authorized users.

## Introduction

Genetic damage is recognized as an enabler of cancer, and exposure to genotoxic substances is an important human health issue (Hanahan and Weinberg 2011). More specifically, exposures to genotoxicants can contribute to the accumulation of mutations in critical genes, such as tumour suppressor or proto-oncogenes (Fearon 1997; Hemminki et al. 2000; Wang et al. 2012; Kucab et al. 2015, 2016), and this can lead to sustained proliferative signalling, replicative immortality, and evasion of apoptosis and growth suppressors. Although genetic damage can occur and accrue spontaneously through endogenous damage and errors in replication, it is also induced by exposures to environmental genotoxicants. Recent high-profile publications have re-emphasized the importance of extrinsic factors such as environmental exposures in determining cancer risk (Alexandrov et al. 2016; Wu et al. 2016).

The pervasive nature of environmental genotoxicants, the recognition in the 1970s and early 1980s that humans are invariably exposed to environmental mutagens, and the established empirical and mechanistic links between genetic damage and carcinogenesis, initiated formal chemical screening programs in Canada, the United States, Japan, and Western Europe (MacGregor et al. 2015a). Importantly, chemical screening for genetic toxicity is now also motivated by recognition that cancer is not the only consequence of somatic mutations. Recent research has demonstrated links between in utero (geno)toxicant exposures and neurodegenerative diseases (Modgil et al. 2014), reproductive defects (Fowler et al. 2008; Mocarelli et al. 2011), child development (Perera et al. 2015) and somatic mosaicism (Erickson 2010; Meier et al. 2016).

Traditional screening for chemically induced genetic toxicity involves high dose exposures followed by binary, qualitative evaluation of the results (i.e., genotoxic or not); little quantitative data analysis is conducted. This screen-and-bin approach is based on the assumption that it is not possible to identify a level below which effects are expected to be negligible (i.e., indistinguishable from the ever-present background); and, moreover, that the relationships between exposure and genotoxic effect are linear to zero dose (Health Canada 1994; MacGregor et al. 2015a; Pottenger and Gollapudi 2010). This assumption is increasingly being challenged, and it is now recognized that quantitative analyses of dose-related effects can be used to identify a dose below which the measured effect level is not significantly elevated (Gollapudi et al. 2013; Johnson et al. 2014; MacGregor et al. 2015b; Speit et al. 2000). However, due to the historical bias of examining effects at high doses, with few dose groups, determination of an accurate point-of-departure (PoD) (i.e., exposure level associated with a pre-defined level of effect) is complicated by the necessity to extrapolate below the tested doses.

The international genetic toxicology community recently examined the use of quantitative dose–response analyses for the determination of genetic toxicity PoD values, as well as their use to determine human exposure limits for regulatory decision-making (Gollapudi et al. 2013; Johnson et al. 2014). Although several methods can be used to analyze dose–response data and generate genotoxicity PoD metrics, the aforementioned works of Gollapudi et al. (2013), Johnson et al. (2014), and MacGregor et al. (2015a) expressed a distinct preference for the benchmark dose (BMD)-approach. The BMD method is a statistical approach for quantitative analysis of dose–response data, whereby a benchmark response (BMR) is selected as a pre-defined effect level (e.g., 10% greater than control) and the statistically determined BMD is the best estimation of the dose that will elicit this BMR. The approach is flexible with regard to study design (e.g., number of dose groups and number of animals) and critically, the 90% upper and lower confidence limits on the BMD (i.e., the BMDU and BMDL) enable statistically rigorous BMD comparisons (Crump 1984; MacGregor et al. 2015a; Slob 2002).

The BMD approach can be used for a variety of purposes, including sensitivity ranking, evaluation of mode-of-action (MOA) hypotheses, investigation of empirical relationships between genetic toxicity endpoints, and determination of exposure limits. For example, we recently used BMD 90% confidence intervals (CIs) to conduct potency rankings across compounds and exposure regimes, and sensitivity rankings across tissues and cell types (Wills et al. 2016a, b). The BMD approach for potency or sensitivity ranking is superior to comparisons based on the no or lowest observed genotoxic effect level (NOGEL or LOGEL), since the PoD derived (i.e., the BMD) is not restricted to the selected dose groups. Moreover, plotting BMD 90% CIs, and arranging them by mid-point, provides a visually intuitive way to compare BMDs and evaluate trends across compounds, sex, cell type, tissue, exposure regime and other covariates.

The BMD approach has also proved useful for the demonstration of quantitative relationships between genotoxicity endpoints. Such demonstrations, which could investigate relationships between different endpoints or between tissues for a single endpoint, can be used to support the involvement of specific key events in the MOA to the adverse outcome (e.g., cancer). For example, Soeteman-Hernández et al. demonstrated correlations between in vivo genotoxic potency (i.e., BMD for induced mutant or micronucleus frequency) and carcinogenic potency (Hernández et al. 2011; Soeteman-Hernandez et al. 2016). However, there is a paucity of data to investigate quantitative relationships between several well-understood key events, such as DNA adduct formation, mutations, and chromosomal damage that are triggered by genotoxicants in their carcinogenic MOAs.

Although it is well accepted that a sub-set of DNA adducts will contribute to mutation formation (Hemminki et al. 2000; Hemminki 1993), there is an acute need for research examining quantitative relationships between adduct frequency and mutation frequency, especially at low doses (Hemminki et al. 2000; Sander et al. 2005). The lack of such analyses quantitatively linking in vivo genetic toxicity (e.g., frequency of DNA adducts) with in vivo mutagenicity, across numerous somatic tissues, is likely due to the difficulty of making all the required measurements. Somatic mutations in vivo are rare and they infrequently elicit phenotypic changes; thus chemically induced changes in mutant frequency (MF) are notoriously difficult to detect; moreover, measurement of DNA damage and MF in the same animals constitutes a significant challenge. Although endogenous mutation detection systems do exist, they are either laborious, and hence, infrequently used (e.g., *Hprt* mutations), or restricted to haematopoietic tissues (e.g., *Pig*-*a*). Transgenic rodents (TGRs), which employ a bacterial target gene in a shuttle vector that can readily be recovered from genomic DNA, provide a convenient way to determine in vivo MF in any tissue (Lambert et al. 2009).

In this study, we used the prototypical genotoxic carcinogen benzo[a]pyrene (BaP), four well-characterized genetic toxicity endpoints, and the BMD approach, to scrutinise responses at low doses, rank potencies across tissues, and compare responses across endpoints. To permit simultaneous determination of several endpoints within the same animal, we employed the MutaMouse, a CD2F1 transgenic mouse containing a *lacZ* target in a lambda shuttle vector (Gossen et al. 1989). We used an extended range of BaP doses (i.e., 11 in total) to examine effects at doses below the region where significant increases in genotoxicity are observed. Although admittedly atypical, the study design employed herein affords improved BMD precision and a concomitant opportunity to robustly compare responses across tissues and endpoints; moreover, to examine empirical relationships between responses of functionally related endpoints (e.g., induced DNA damage and mutations).

## Materials and methods

### Animal exposures and tissue collection

We selected eleven doses of BaP (CAS # 50-32-8, purity ≥ 96%; Sigma-Aldrich Canada, Oakville, ON, Canada), with the top doses based on previous work by our group (Lemieux et al. 2011). The selected doses, 0, 0.10, 0.20, 0.39, 0.78, 1.56, 3.13, 6.25, 12.50, 25.00, and 50.00 mg BaP/kg body weight (BW)/day, were delivered in highly refined olive oil (Sigma-Aldrich). Adult male MutaMouse specimens (12–13 weeks old) were maintained as described previously (Long et al. 2016). There were 7 animals in each dose group, with 14 animals in the vehicle control group (84 animals total). Mice received BaP or olive oil by oral gavage at 0.005 ml/g body weight daily for 28 days. Two days after the final dose, blood was collected from the facial vein for MN analysis. A 3-day sampling time was employed for the transgene endpoint (OECD 2013), and was also employed for the *Pig*-*a* endpoint. Mice were anesthetised with isoflurane gas and blood was collected via cardiac puncture for scoring *Pig*-*a* MF. Additionally at this time, blood was collected from 4 positive control mice that were administered 80 mg ENU/kg BW i.p., and four vehicle control mice that were administered phosphate buffer as single i.p. injection 3 weeks prior to blood collection. Mice were euthanized by cervical dislocation. Mice were bred, maintained, and treated in accordance with the Canadian Council for Animal Care Guidelines and Health Canada’s Animal Care Committee. Bone marrow, liver, lung, small intestine, and glandular stomach were preserved as described previously (Long et al. 2016). Kidney, spleen, and bladder were flash frozen in liquid nitrogen. All tissues were stored at – 80 °C.

Preliminary data for bone marrow, liver, small intestine, glandular stomach, and lung for the *lacZ* MF endpoint only were previously published in Wills et al. (2016b); here the dataset is expanded with a further two tissues (i.e., kidney and spleen) for *lacZ*, and three additional endpoints.

### Peripheral blood micronucleus assay

MicroFlow^®^-BASIC kits (Litron Laboratories, Rochester, NY, USA) were used to prepare blood cells for enumeration of micronucleated reticulocytes (MN-RET) and normochromatic erythrocytes (MN-NCE) at Litron Laboratories, as previously described (Long et al. 2016). Frozen, coded samples were shipped on ice to Litron Laboratories for scoring by flow cytometry using a three-colour labelling method as previously described (Dertinger et al. 2004).

### Pig-a mutant scoring in peripheral blood

Following blood collection via cardiac puncture, 100 µl of blood was immediately transferred to a 0.5 mL K2-EDTA-coated microtainer (VWR). Whole blood samples were placed in an ExactPak shipping container and shipped on ice overnight to Litron Laboratories. Flow cytometric scoring of *Pig*-*a* MF was conducted at Litron via MutaFlow^®^ Kit reagents according to their published immunomagnetic enrichment method (Dertinger et al. 2011), with modifications for scoring in mouse (Labash et al. 2016). Based on these analyses, the number of *Pig*-*a* mutant cells per million were calculated for RETs as well as total red blood cells (RBCs).

### DNA extraction

Bone marrow, glandular stomach, liver, small intestine, and lung were prepared for an overnight digestion in lysis buffer as described previously (Long et al. 2016). Spleen, kidney, and bladder were prepared for overnight lysis and genomic DNA extraction as follows: approximately ¼ of the spleen, ½ of a kidney, or the entire bladder was defrosted on ice and minced into small pieces. The minced tissue was transferred to a tube containing 5 ml lysis buffer [1 mM Na_2_EDTA, 100 mM NaCl, 20 mM Tris–HCl, pH 7.4, 1% SDS (w/v)] and incubated overnight at 37 °C with gentle shaking. Genomic DNA was isolated from lysed tissue using a phenol/chloroform extraction procedure described previously (Douglas et al. 1994; Vijg and Douglas 1996). Isolated DNA was dissolved in 50–100 µl TE buffer (10 mM Tris pH 7.6, 1 mM EDTA) and stored at 4 °C until use.

## ^32^P-postlabelling for DNA adduct analysis

Bulky DNA adduct formation was analysed in DNA samples from liver, lung, bone marrow, glandular stomach, small intestine, spleen, kidney, and bladder via the nuclease P1 enrichment version of the thin-layer chromatography ^32^P-postlabelling assay (Phillips and Arlt 2014). The procedure was performed as described previously (Krais et al. 2016; Wohak et al. 2016), and results are expressed as DNA adducts/10^8^ nucleotides.

### Positive selection for lacZ mutants

The PGal (phenyl-β-d-galactoside) positive selection assay was carried out for the analysis of *lacZ* MF in DNA samples from spleen and kidney as previously described (Gossen et al. 1992; Lambert et al. 2005; Vijg and Douglas 1996). MF was calculated as the ratio of mutant plaque forming units (pfu) to total pfu. The *lacZ* MF could not be scored in bladder as there was insufficient DNA remaining following DNA adduct analysis. The *lacZ* MF data for bone marrow, glandular stomach, small intestine, liver, and lung was recently published in Wills et al. (2016b), and these data were included in the analyses presented below.

### Statistical analysis

Statistical analysis for the determination of a treatment effect for all endpoints was carried out in SAS v.9.1 (SAS Institute, Cary, NC, USA) by applying a Type 3 Chi-square analysis and employing a Poisson regression, in the same manner as described previously (Long et al. 2016). Post-hoc custom contrasts based on the asymptotic Chi-square distribution of the likelihood ratio statistic were conducted to compare each dose group with the control.

### Benchmark dose modelling

BMD analyses were conducted using the PROAST software (version 50.9—http://www.proast.nl). Dose–response data were analysed using a family of nested exponential models (Slob 2002) recommended by the European Food Safety Authority for the assessment of continuous data (EFSA 2009). PROAST uses the likelihood ratio test to select the optimal model, with increasingly complex models using additional parameters only accepted if the difference in log-likelihood exceeds the critical value of *p* < 0.05. The BMR selected for the current analyses (i.e., 100% or a two-fold increase in the response relative to control), was chosen as it is commonly used for the assessment of genotoxicity data, and because it tended to lie within the range of experimental observation in the datasets herein as was thus ‘optimal’ for deriving CIs for sensitivity comparison (Wills et al. 2016a). The BMDL and BMDU values represent the lower and upper bounds of the two-sided 90% CI of the BMD, respectively, with the difference between the BMDU and BMDL defining the uncertainty in the BMD estimate and, therefore, its precision. As employed in previous work, CI plots arranged by the geometric midpoint of the BMDL-BMDU interval were utilised to permit robust sensitivity comparisons that account for estimation uncertainty (Bemis et al. 2016; Wills et al. 2016a).

To evaluate the relationship between *lacZ* BMDs and adduct BMDs, the visual approach used by Bemis et al. (2016) and Soeteman-Hernández et al. (2016) was employed. This approach simply draws a line with unity slope through the double-log plot; the unity slope translates into a proportional relationship between the endpoint BMDs on the original axes scales.

## Results

No overt signs of toxicity (i.e., body weight change, liver somatic index) were observed in any dose group, including controls. A significant level of induction of each genetic damage endpoint was observed in all tissues examined, although the responses occurred at different dose levels and the magnitude of the response varied across endpoints. Using PROAST, we employed the exponential model family to derive the BMD_100_ and accompanying 90% CIs (i.e., the BMDL and BMDU values). The BMD_100_, BMDL, BMDU, along with the BMDU-BMDL ratio for each tissue and endpoint are summarised in Supp. Table I, and BMD model fits for each analysis are presented in Supp. Figure 1a–d. The CIs (i.e., range between the BMDL to BMDU) bounding the BMD_100_ values were used for comparative evaluations of tissue sensitivities.

### DNA adduct frequency

**Fig. 1:**
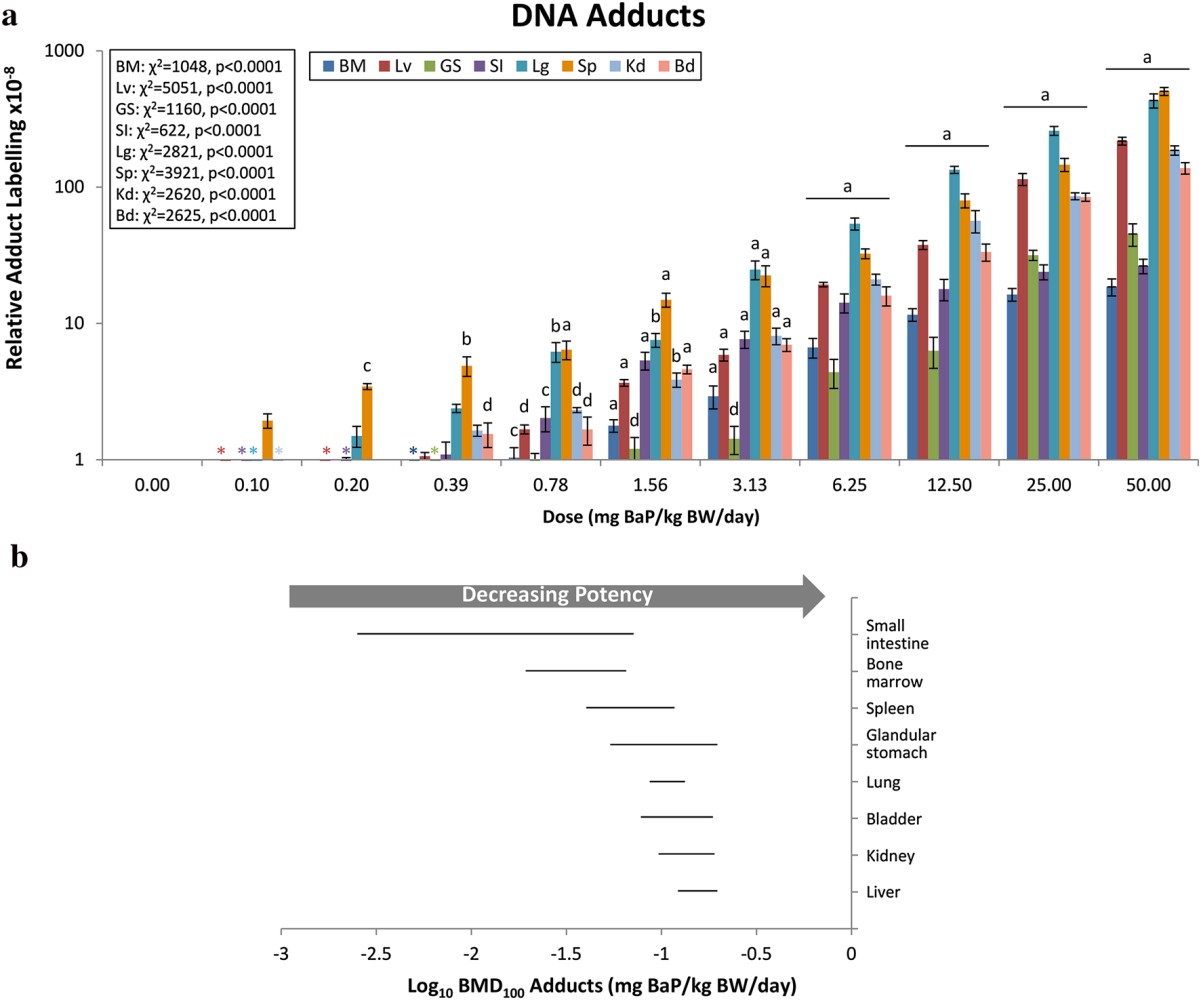
The frequency of BaP-induced DNA adducts was evaluated in 8 tissues. **a** BaP dose–response data (± standard error) for DNA adducts plotted using a log_10_ y-axis for better visualisation of the responses across all doses. Statistical results for the overall dose–response relationship are presented for each tissue. The level of significance for the custom contrast results for each dose vs. control are indicated as follows: *a* = *p* < 0.0001; *b* = *p* < 0.001; *c* = *p* < 0.01; *d* = *p* < 0.05. *BM* bone marrow, *Lv* liver; *GS* glandular stomach; *SI* small intestine; *Lg* lung; *Sp* spleen; *Kd* kidney; *Bd* bladder. Asterisk indicates where responses were below 1, and thus are obscured on a log_10_ scale. Supp. Figure 3 shows dose–response data plotted on a linear scale with a restricted *y*-axis to visualise the low-dose responses. **b** BMD_100_ values and two-sided 90% CIs (i.e., the range between the BMDL and BMDU) determined using the exponential model for DNA adducts. BMDs can be considered different where CIs do not overlap

Using an external BaP-diol-epoxide-DNA standard (Phillips and Castegnaro 1999), the major DNA adduct detected was identified as dG-*N* ^2^-BPDE (10-(deoxyguanosin-*N* ^2^-yl)-7,8,9-trihydroxy-7,8,9,10-tetrahydro-BaP), with BaP-DNA adducts being detected in all seven tissues examined (Supp. Figure 2). The first dose in which we observed a significant increase in adduct frequency over control (i.e., the LOGEL) was 0.20 mg/kg BW/day for spleen, 0.39 mg/kg BW/day for bladder, 0.78 mg/kg BW/day for bone marrow, liver, small intestine, lung, and kidney, and 1.56 mg/kg BW/day for glandular stomach (Fig. 1a; Supp. Figure 3). The highest fold change increase in DNA adduct levels over control was observed in spleen (506-fold), followed by lung (433-fold), liver (219-fold), kidney (187-fold), bladder (139-fold), glandular stomach (46.3-fold), small intestine (27.4-fold), and bone marrow (19.6-fold).

To visualize the difference in tissue sensitivities, the BMD CIs for each tissue were plotted using a log_10_-scale (Fig. 1b). In this way, tissues with non-overlapping BMD CIs can be sequentially ranked by sensitivity, with decreasing sensitivity shown from left to right (i.e., lowest to highest BMD). This method for the illustration of sensitivity trends is superior to the common approach of simply listing the BMD/BMDL/BMDU values in a table (Supp. Table I), as upon inspection of Fig. 1b it is immediately apparent that the sensitivity order is: small intestine/bone marrow/spleen/glandular stomach/lung/bladder/kidney/liver. Due to the relatively large BMD CI for some tissues (e.g., 5 of 8 tissues had BMDU-BMDL ratios over 2, Supp. Table I), there is some overlap in the BMD range of all adjacent tissues. However, it is still possible to obtain mechanistic information from these results; and, moreover, conclude that the sensitivity of small intestine and bone marrow are significantly higher than lung, bladder, kidney, and liver. It is also interesting to note that the sensitivity order obtained via BMD-modelling is not the same as the order of tissues according to the level of induced response (i.e., maximum fold-change over control), nor the order determined using lowest significant dose (i.e., the LOGEL).

### lacZ mutant frequency

**Fig. 2:**
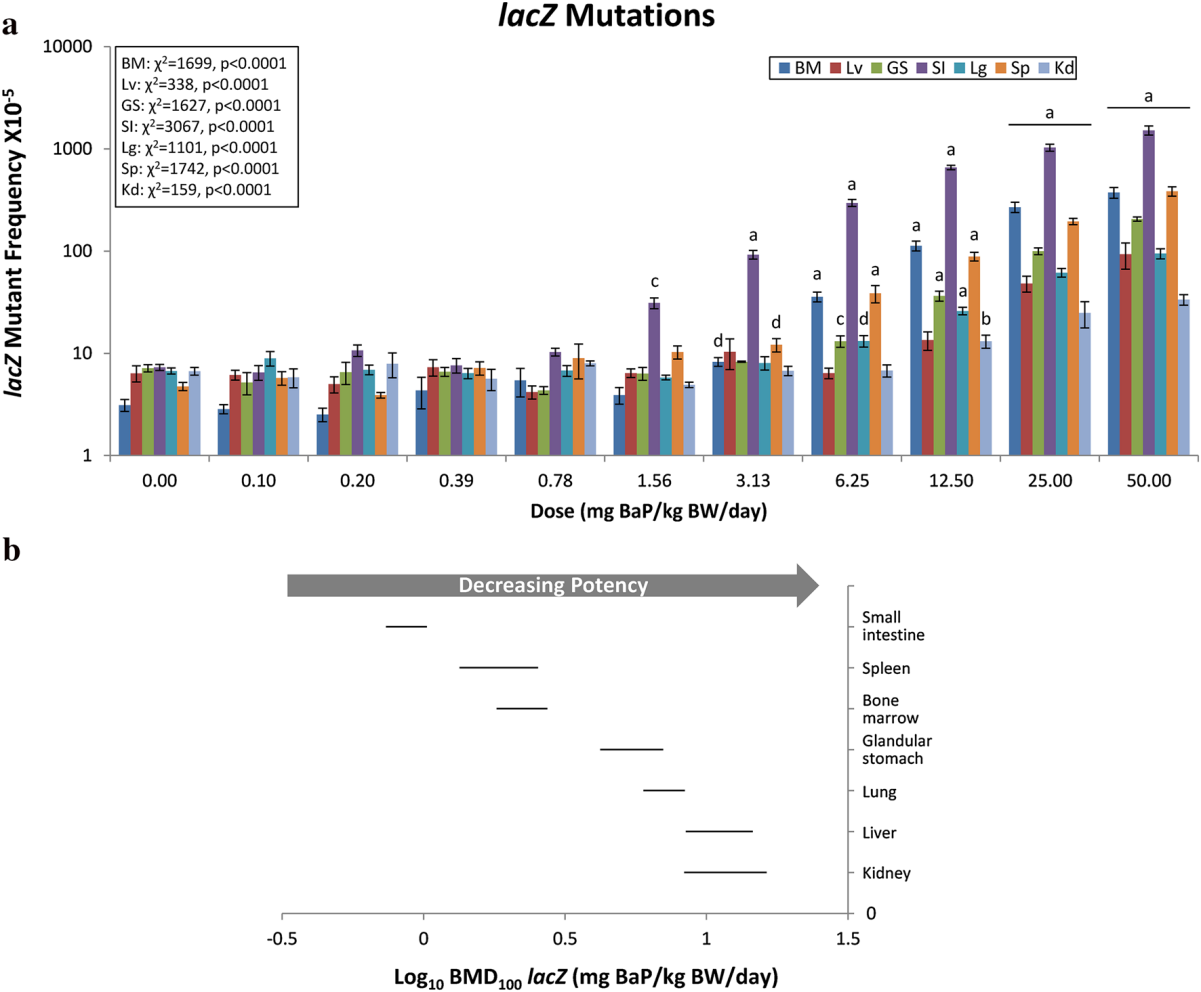
The frequency of BaP-induced *lacZ* mutants was evaluated in seven tissues. **a** BaP dose–response data (± standard error) for *lacZ* mutations, plotted using a log_10_ y-axis for better visualisation of the responses across all doses. Statistical results for the overall dose–response relationship are presented for each tissue. The level of significance for the custom contrast results for each dose vs. control are indicated as follows: *a* = *p* < 0.0001; *b* = *p* < 0.001; *c* = *p* < 0.01; *d* = *p* < 0.05. *BM* bone marrow; *Lv* liver; *GS* glandular stomach; *SI* small intestine; *Lg* lung; *Sp* spleen; *Kd* kidney. **b** BMD_100_ values and two-sided 90% CIs (i.e., the range between the BMDL and BMDU) determined using the exponential model for *lacZ* mutations. BMDs can be considered different where CIs do not overlap

A significant induction in *lacZ* MF was observed in all tissues examined, with LOGELs of 1.56 mg/kg BW/day for small intestine, 3.13 mg/kg BW/day for bone marrow and spleen, 6.25 mg/kg BW/day for lung and glandular stomach, 12.5 mg/kg BW/day for kidney, and 25 mg/kg BW/day for liver (Fig. 2a). The highest fold change increase over control was observed in small intestine (208-fold), followed by bone marrow (120-fold), spleen (81.0-fold), glandular stomach (28.6-fold), liver (14.6-fold), lung (14.1-fold), and kidney (5.0-fold). As Fig. 2b illustrates, there were considerable differences in BMDs across tissues, with the sensitivity order as follows: small intestine > spleen or bone marrow > glandular stomach or lung > liver or kidney. Despite minor differences, it is apparent that this sensitivity trend is similar to that observed for DNA adduct frequency. However, for the *lacZ* endpoint the BMDs were more precise (i.e., all BMDU-BMDL ratios below 2; Supp. Table I), which resulted in fewer overlapping CIs. As a result, it is possible to distinguish *lacZ* sensitivity values between additional tissues, and in fact, to visualise three discrete groupings of tissues (i.e., small intestine > bone marrow/spleen > kidney/liver/lung/stomach). In this case, the sensitivity order is fairly similar to the order according to induced response and LOGEL.

### Pig-a mutant frequency in peripheral blood

**Fig. 3:**
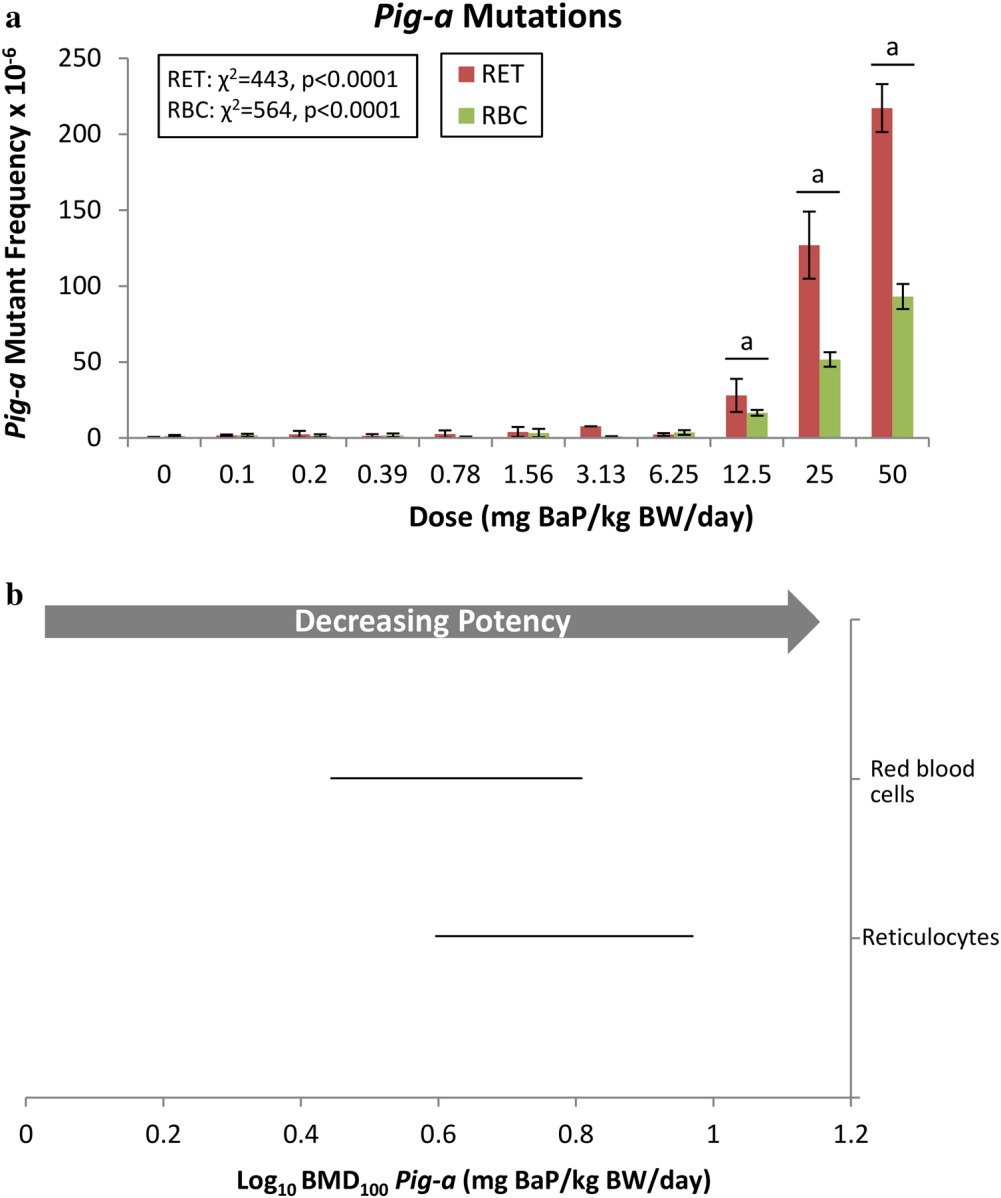
The frequency of BaP-induced *Pig*-*a* mutant phenotype was evaluated in peripheral blood. **a** BaP dose-response data (± standard error) for *Pig*-*a* mutations. Statistical results for the overall dose–response relationship are presented for both cell populations. The level of significance for the custom contrast results for each dose vs. control are indicated as follows: *a* = *p* < 0.0001; *b* = *p* < 0.001; *c* = *p* < 0.01; *d* = *p* < 0.05. *RET* reticulocyte; *RBC* red blood cell. **b** BMD_100_ values and two-sided 90% CIs (i.e., the range between the BMDL and BMDU) for *Pig*-*a* mutations for both cell populations Where CIs overlap tissue responses cannot be considered different

*Pig*-*a* mutant phenotype frequency was examined in both RETs and RBCs. A significant positive increase was observed in both cell populations, with the first significant response (i.e., the LOGEL) appearing at 12.5 mg/kg BW/day in both RETs and RBCs (Fig. 3a). The fold-increase over control observed for RBCs (69.7-fold) was only 1/5 as high as for RETs (385.5-fold). The BMD CIs for RETs and RBCs overlapped (Fig. 3b), therefore, a sensitivity order could not be established. This is interesting given the large difference in response magnitudes; however, the trend across these two cell populations is analogous to the observed cross-tissue trend in sensitivity to adduct formation (i.e., overlapping BMD ranks). In this case, issues related to the detection of the *Pig*-*a* MF resulted in reduced sample size that likely contributed to increases in the BMD CIs. In fact, the *Pig*-*a* BMDU-BMDL ratio is over 2 for both cell populations, whereas the *lacZ* BMDU-BMDL ratio is below 2 for all tissues examined.

### Percent micronuclei in red blood cells

**Fig. 4:**
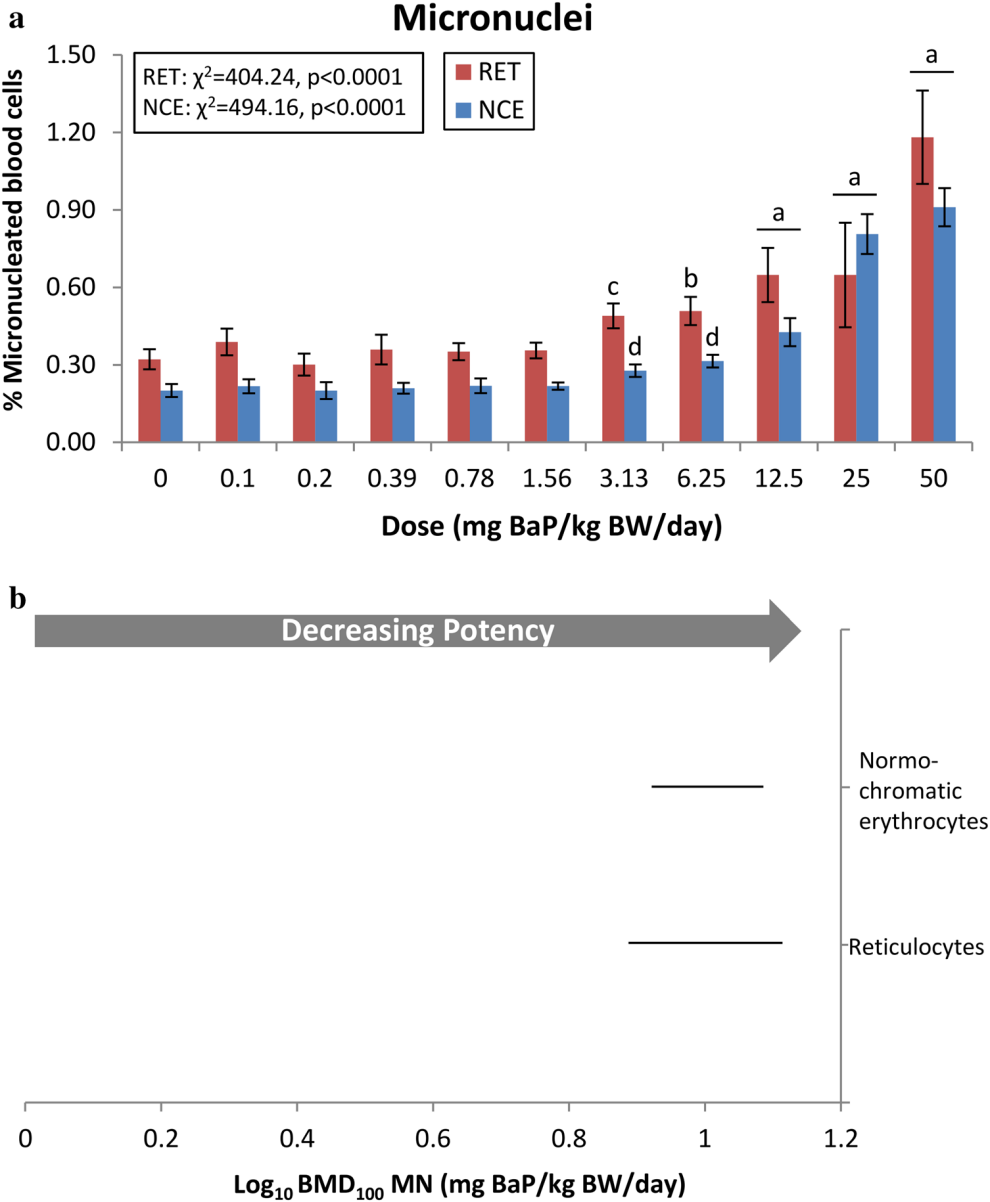
The frequency of BaP-induced MN was evaluated in peripheral blood. **a** BaP dose-response data (± standard error) for micronuclei. Statistical results for the overall dose–response relationship are presented for both cell populations. The level of significance for the custom contrast results for each dose vs. control are indicated as follows: *a* = *p* < 0.0001; *b* = *p* < 0.001; *c* = *p* < 0.01; *d* = *p* < 0.05. *RET* reticulocyte; *NCE* normochromatic erythrocyte. **b** BMD_100_ values and two-sided 90% CIs (i.e., the range between the BMDL and BMDU) for % micronuclei for both cell populations. Where CIs overlap tissue responses cannot be considered different

The frequencies of micronucleated RETs and NCEs were examined to assess BaP-induced chromosomal damage. A significant increase in the percent micronucleated cells was observed in both RETs and NCEs at 3.13 mg/kg BW/day (Fig. 4a). A slightly higher fold-change increase over control was observed in RETs (4.5-fold), in comparison with NCEs (3.7-fold). The ranges between the BMDL and BMDU overlapped (Fig. 4b), therefore, a sensitivity order could not be determined. The ratio of BMDU to BMDL was well below 2, indicating that BaP is similarly clastogenic in both cell populations.

### Comparisons of endpoint-specific BMDs for hematopoietic tissues

**Fig. 5:**
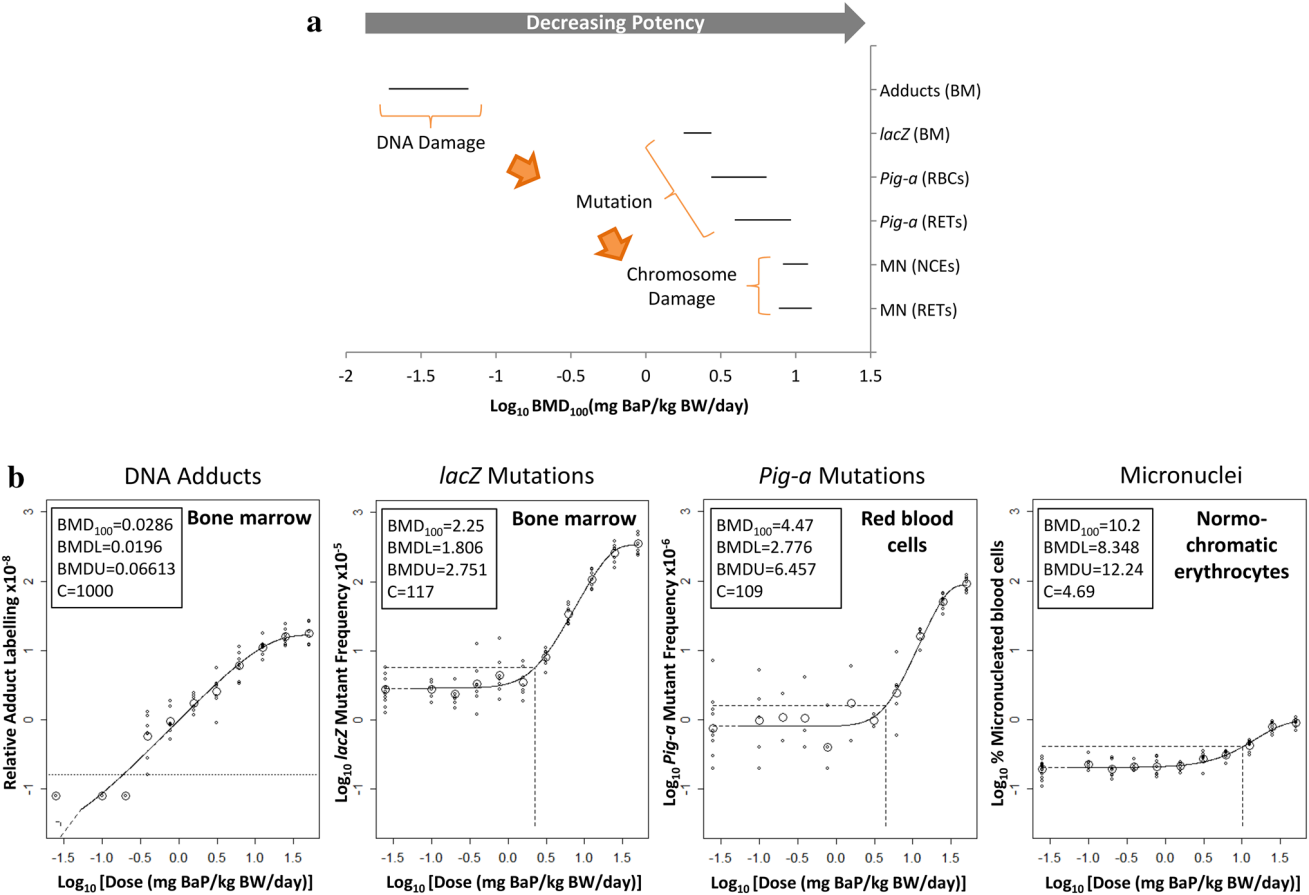
The observed pattern of hematopoietic tissue BMDs showing important differences in the maximum response across endpoints. **a** BMD_100_ values and two-sided 90% confidence intervals (i.e., the range between the BMDL and BMDU) for each endpoint in hematopoietic tissue. **b** Illustration of fitted functions on a fixed y-axis showing variations in maximum response, indicated by model parameter ‘*c*’ (inset), across endpoints. The horizontal dashed lines indicate the BMR on the *Y*-axis, and the vertical dashed lines indicate the BMD on the *X*-axis

Finally, we compared sensitivity across each endpoint for hematopoietic tissues. To do this, the 90% CIs for each hematopoietic tissue BMD_100_ were plotted together for visual comparison across the endpoints examined (Fig. 5a). The figure shows a progression of BMD_100_ values across endpoints from DNA adducts (i.e., lowest BMD), to *lacZ* and *Pig*-*a* mutants at doses approximately 25- to 90-fold higher, and finally, to chromosome damage at doses 2- to 4.5-fold higher than the mutant endpoints and 150- to 350-fold higher than for DNA adducts (Supp. Table I). Thus, the observed pattern of BMDs in hematopoietic tissues seems consistent with the sequence of key events leading from genetic damage to cancer.

Although Fig. 5a supports the contention that sequential genotoxic events precede tumor formation (Fukushima et al. 2016; MacGregor et al. 2015b) the manner in which the data are presented raises fundamental questions about the validity of such cross-endpoint comparisons. Figure 5b uses identical fixed y-axes to compare the modelled dose–responses for each endpoint in hematopoietic tissue (RBCs only for *Pig*-*a*, NCEs only for MN). By plotting the responses on the same y-axis scale, it becomes readily apparent that the inducible range in response varies considerably across endpoints (e.g. a small dynamic range in inducible response is particularly noticeable for the micronucleus endpoint). Additionally, model parameter ‘c’ displayed in the inset, which is the model-determined estimate of the maximum response, shows nearly a 10-fold reduction between the DNA adduct and MF endpoints, and an approximately 25-fold reduction between the MF and micronucleus endpoints (Fig. 5b).

### Empirical relationship between genotoxic sensitivity and mutagenic sensitivity

**Fig. 6:**
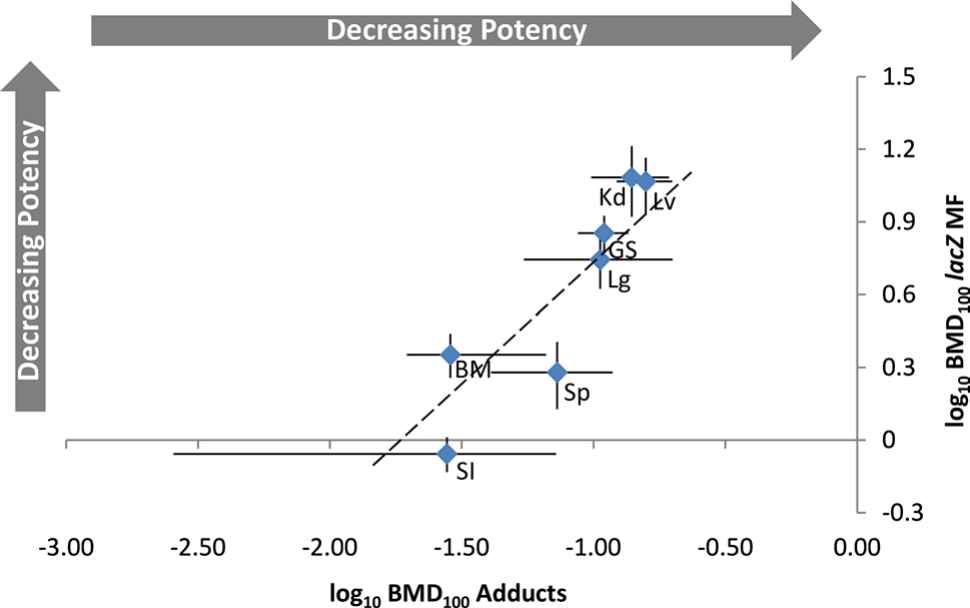
The empirical relationship between genotoxic sensitivity and mutagenic sensitivity of BaP (i.e., BMD_100_ values and two-sided 90% CIs for the DNA adduct endpoint vs. tissue-matched BMD_100_ values and two-sided 90% CIs for the* lacZ* mutation endpoint). A line with unity slope is indicated, which, on a double-log plot, demonstrates proportionality between the responses. *BM* bone marrow; *Lv* liver; *GS* glandular stomach; *SI* small intestine; *Lg* lung; *Sp* spleen; *Kd* kidney

When visually comparing the dose–response data for DNA adduct frequency (Fig. 1a) with that for *lacZ* MF (Fig. 2a), there is no apparent empirical relationship between the tissue-specific induced damage levels. To examine the empirical relationship between these two endpoints, tissue-matched BMD_100_ values, and their associated 90% CIs, were plotted against each other. To quantitatively scrutinise the correlation on the double-log axes, a diagonal line with unity slope (i.e., slope = 1) was overlaid on the plotted data (Fig. 6). If the BMDs for the various tissues scatter randomly around the unity line, and the unity line appears to be a good representation of the data, this shows that the mutant and adduct BMDs are *proportionally* related on the original scales. The analysis, which is shown in Fig. 6, indeed demonstrated a proportional relationship between genotoxic sensitivity (i.e., induction of DNA adducts), and mutagenic sensitivity (i.e., induction of *lacZ* mutants) across seven tissues. Critically, this proportionality suggests that, despite large differences in cross-tissue BMDs for a given endpoint, tissue-specific conversion of adducts to mutations is likely consistent.

As cellular replication is a necessary factor for mutation fixation, we also wanted to investigate the influence of cellular proliferation on the conversion of DNA adducts to mutants. As part of our standard experimental protocol, we evaluated proliferation rate, as Ki-67 index, in liver, lung, and small intestine (Supp. Methods). The Ki-67 index plotted against the ratio of DNA adduct BMD to *lacZ* MF BMD for these three tissues (Supp. Figure 4), is positively correlated. Although this correlation is based on only three tissues, it supports the notion that cellular proliferation rate affects the tissue-specific conversion of BaP-induced DNA adducts to mutations.

## Discussion

The quantitative assessment of genetic toxicity data is a rapidly advancing field, and the emerging application of the BMD-approach to derive PoDs has well-described advantages for regulatory decision-making and the protection of human health. Here, we present an advanced approach for BMD-modeling that allows for robust cross-tissue sensitivity ranking, thereby contributing important quantitative support for tissue-specific genotoxic effects. More specifically, this study used the BMD-approach to model BaP-induced in vivo genetic toxicity dose–response relationships across several tissues and an expanded dose range, and derived BMD_100_ values and their associated 90% CIs for each tissue/endpoint combination. We subsequently employed the BMD CIs to conduct sensitivity ranking across eight tissues for DNA adducts, seven tissues for *lacZ* mutants, and 2 peripheral blood cell populations for both *Pig*-*a* MF and MN. As discussed below, examinations of tissue rankings for each endpoint, and empirical relationships between the endpoints, permitted general statements about the fundamental nature of the endpoints examined and the mechanistic relationships between the damage types.

It is well recognized that BaP primarily induces tumors via a genotoxic mode-of-action; specifically, via the conversion of bulky DNA adducts to point mutations that confer the hallmark properties of a cancerous cell (Hanahan and Weinberg 2011; IARC 2010b). Although not the primary mechanism, BaP is also known to induce micronuclei, as double strand breaks may be formed as a result of replication fork stalling and collapse (Bi et al. 2005). Following oral exposure, BaP has been shown to induce carcinomas in the oral cavity, forestomach, liver, and small intestine of male and female Wistar rats, and kidney, mammary, and skin carcinomas in male Wistar rats (Kroese et al. 2002). Oral administration to various mouse strains resulted in tumours in lymphoid and haematopoietic tissues, as well as in lung, forestomach, liver, esophagus, and tongue (IARC 2012). BaP also caused forestomach carcinomas, malignant lymphomas in lymphatic organs including the spleen, and bronchiolar-alveolar hyperplasia in the lung of male MutaMouse specimens orally exposed for only 5 days (Hakura et al. 1998). It is, therefore, not unexpected that we were able to detect significant increases in BaP-induced genotoxicity for all four endpoints in all tissues/cell populations examined, namely DNA adducts and *lacZ* mutants in bone marrow, glandular stomach, small intestine, liver, lung, kidney, spleen, and bladder (adducts only); as well as *Pig*-*a* MF and chromosome damage in peripheral blood cells. With respect to DNA damage, we were able to detect a significant increase in DNA adducts at 0.20 mg BaP/kg BW/day, the lowest oral dose used to date to investigate the in vivo genotoxicity of BaP. This robust genotoxic response demonstrates the utility of the MutaMouse TGR assay for simultaneously investigating several, complementary genetic toxicity endpoints across numerous tissues.

Ranking sensitivity across the measured tissues lends support for mechanistic understanding of the toxicological properties of the compound evaluated. In this study, we observed that the *Pig*-*a* and MN BMD CIs overlapped for RETs and RBCs/NCEs, and, therefore, a sensitivity order could not be established between the two cell populations. This may simply be due to the accuracy of the measurement of these endpoints, with larger CIs being more likely to result in overlap. However, for the MN endpoint in particular, the BMDU-BMDL ratio is below 2 and is quite similar for both cell populations, therefore, alternatively, this may indicate that RETs and NCEs are equally sensitive to MN induction by BaP. Therefore, that there may be comparable utility in examining either cell population for study designs that involve protracted exposures, such as the one conducted here. Our novel approach to data analysis makes a direct comparison of our results with other publications using these endpoints difficult. However, in a recent paper we employed the BMD method to examine dose–response data from a *Pig*-*a* study examining the genotoxicity of *N*-ethyl-*N*-nitrosourea, and in that case, the BMD CIs for RBCs and RETs overlapped (Wills et al. 2016b), as they did in the current study. Conversely, the CIs for RBCs sampled at different time points were distinct (Wills et al. 2016b), which highlights that sampling time appears to be more important for RBCs than for RETs. Notably, for both the *Pig*-*a* and MN endpoints, far fewer RETs are interrogated than NCEs/RBCs, and this contributes to larger CIs. Additional use of the BMD approach will permit similar cross-cell comparisons for different species, experimental designs, and compounds.

The DNA adduct and *lacZ* MF sensitivity rankings resulted in very similar cross-tissue patterns; however, there was considerably more overlap in CI range across tissues for DNA adducts than for *lacZ* MF. This is likely a result of the fact that the *lacZ* MF data permit determination of more precise BMDs (as demonstrated by the smaller BMDU-BMDL ratio), allowing for greater sensitivity discrimination between tissues.

For both DNA adducts and *lacZ* MF, the cross-tissue pattern in responses is likely controlled by four factors: (1) tissue contact, (2) tissue-specific metabolism and the extent of systemic circulation of the parent compound and activated metabolites, (3) tissue-specific proliferation rate, and (4) tissue-specific differences in DNA repair capacity. As discussed in our recent manuscript (Wills et al. 2016b), the fact that small intestine was the most sensitive tissue for induction of *lacZ* mutants, and similarly, as seen here, for DNA adducts, is consistent with an oral route of exposure and the known metabolic capacity of this tissue. BaP itself is not DNA-reactive. Its major genotoxic pathway (i.e., via cytochrome P450 CYP1A1/1A2/1B1 metabolism) is induced following aryl-hydrocarbon (AhR)-agonism (Shimada et al. 2002; Shimada and Fujii-Kuriyama 2004; Xue and Warshawsky 2005), and leads to the conversion of BaP into the genotoxic metabolite BaP-7,8-dihydrodiol-9,10-epoxide (BPDE). BPDE exerts its mutagenic effect by covalently binding primarily to purines, causing a bulky DNA adduct, which if not repaired prior to DNA replication, can result in a mutation (IARC 2014). Two alternate activation pathways for BaP are also recognized, namely the PAH-radical-cation pathway, which results in depurinating adducts, and the *ortho*-quinone pathway, which can generate covalent DNA adducts or oxidative DNA damage (Penning 2014). However, this study, which employed ^32^P-postlabeling, only examined the frequency of BPDE-type adducts. Although ingested BaP would reach the stomach first, the small intestine is known to have much higher levels of *Cyp1a1* expression (Choudhary et al. 2003; Renaud et al. 2011) and, therefore, absorption by the small intestine would likely result in greater conversion to BPDE, a concomitant increase in damage in this tissue, and a higher sensitivity (i.e., lower BMD) in comparison with stomach (Figs. 1b, 2b**)**. Furthermore, BaP is known to undergo enterohepatic circulation (Miller and Ramos 2001; Ramesh et al. 2004), which causes cycling of BaP and its activated metabolites between the liver and the small intestine via the bile. This would permit re-exposure of the small intestine and liver to BPDE, leading to an increase in bulky adducts and, in turn, mutations. Taken together, the high level of tissue contact and effective metabolism in this tissue would certainly contribute to the high sensitivity in small intestine, as seen here.

As with the site-of-contact tissues, the spleen and bone marrow were quite sensitive to the induction of both DNA adducts and *lacZ* mutants. These are both distal tissues that require systemic circulation of BaP and/or activated metabolites for exposure to occur. BaP is well known to induce significant immunotoxicity in rodent models (Blanton et al. 1986; De Jong et al. 1999; Dean et al. 1983; Ginsberg et al. 1989), which appears to be at least partially mediated by cytotoxicity related to DNA adduct formation (Ginsberg et al. 1989). The lymphatic system, including the spleen, is also a target organ for tumors (lymphomas) in orally exposed MutaMouse (Hakura et al. 1998), and XPA-deficient mice (IARC. 2010a; Van Oostrom et al. 1999). CYP1B1 in the spleen and bone marrow appears to mediate the conversion of BaP to BPDE (Heidel et al. 1998; Uno et al. 2006) with several studies also suggesting the possibility that splenic exposure to BPDE is also occurring as a result of extra-splenic metabolism (Ginsberg et al. 1989; Ginsberg and Atherholt 1990). Specifically, BPDE in the blood can be sequestered by the serum (Ginsberg and Atherholt 1989) and transported to the spleen, where it is released and taken up by splenocytes (White et al. 1994). The results presented herein likely indicate that substantial levels of BaP are available for systemic circulation, and support the high sensitivity observed for both bone marrow and spleen.

The cross-tissue sensitivity ranking for the *lacZ* endpoint also highlights the likely role of tissue-specific proliferation, since the sensitivities for tissues with higher proliferative rates (i.e., small intestine, spleen, bone marrow, glandular stomach) were higher than tissues with slower rates of proliferation (i.e., lung, liver, kidney). For example, the sensitivity of the hepatic *lacZ* response is the lowest (i.e., highest BMD), and this is consistent with a relatively low proliferation rate in comparison with the other tissues examined. In order for bulky adducts to result in sequence changes, DNA replication is a necessary process, therefore, tissues that are not actively undergoing cellular proliferation would accrue fewer mutants in a given period of time, whereas a tissue with a higher mitotic index would be likely to accrue far more mutants. It is known that spleen (Muskhelishvili et al. 2003), bone marrow (White et al. 2017), glandular stomach (Merritt et al. 1997; Ozkan et al. 2001; Radulescu et al. 2010; Snipes 1967), and small intestine (Muskhelishvili et al. 2003; White et al. 2017) have mitotic indices that are at least an order of magnitude higher than those of slower proliferating tissues such as liver (White et al. 2017), lung (Shami and Evans 1992), and kidney (Eldridge and Goldsworthy 1996). These higher replication rates would be expected to contribute to higher mutagenic efficiency (i.e., conversion of stable DNA adducts to mutations), higher mutant responses for a given dose, and a concomitantly lower BMD (i.e., lower sensitivity), which is consistent with our results. In fact, when we plotted the Ki-67 index for liver, lung, and small intestine against the ratio of the BMD_100_ of DNA adducts to BMD_100_ of *lacZ* mutations (Supp. Figure 4), we obtained a significant positive correlation. Taken together, the data presented here and in the literature highlight the importance of tissue-specific cellular proliferation in the conversion efficiency of stable DNA adducts to mutations.

The tissue-specific difference in DNA-repair capacity is also likely to contribute to the observed sensitivity pattern across tissues. Recent in vitro studies of potent alkylating agents have demonstrated that inactivation of O^6^-methylguanine DNA methyltransferase (MGMT) can shift the PoD for mutation and chromosome damage to the left (i.e., more potent) (Thomas et al. 2013; Zair et al. 2011), whereas murine overexpression of the same repair pathway moves the PoD for tumor formation induced by alkylating agents significantly to the right (i.e., less potent) (Becker et al. 2014). Although differences in repair capacity across various tissues have not been fully explored, it is likely that tissue-specific differences in the presence and activity of certain DNA repair genes will affect the rate of conversion of bulky adducts to mutations. This is consistent with the aforementioned work of Thomas et al. (2015), which noted that the four key proteins involved in nucleotide excision repair (i.e., the pathway associated with BPDE removal) can be induced by genotoxic stress.

The direct comparison of BMDs across endpoints is an approach used to permit ranking of mechanistically sequential endpoints (Fukushima et al. 2016; Moffat et al. 2015; Thomas et al. 2007). The approach, which is borrowed from chemical risk assessors’ practice of employing the most sensitive endpoint (i.e., lowest NOAEL) for regulatory decision-making, involves ordering endpoints that represent a series of plausible key events, and then comparing PoD metrics across these endpoints. When the BMD_100_ values calculated here are compared across all hematopoietic tissue endpoints (Fig. 5a), the observed BMD pattern is consistent with the sequence of key events leading from genetic damage to cancer. At first glance this is intuitive, as we expect DNA adducts to occur at a dose that is only limited by test article absorption, metabolism, and distribution, and tissue exposure to activated metabolites. Adducts may be repaired, however; and only when this response becomes overwhelmed, presumably at a higher dose, would we expect to see mutations and/or chromosome damage. Thus, the BMD for the next endpoint in the series (i.e., mutations) would be expected to be equal to or higher than that for adducts (Meek et al. 2014), as we found here with the BMD for *lacZ* mutants in bone marrow, which preceded that for *Pig*-*a* MF. The lower BMD for *lacZ* mutants is likely a result of the fact that *lacZ* is a multi-copy silent transgene (Shwed et al. 2010), whereas *Pig*-*a* is an active, single copy endogenous gene (Phonethepswath et al. 2008). As such, *lacZ* transgene DNA is not subject to transcription-coupled repair (Lambert et al. 2005), resulting in a higher MF for a given dose (i.e., lower BMD) in comparison with an endogenous gene like *Pig*-*a*. Finally, MN are the result of a relatively narrow range of damage types (i.e., double strand breaks or whole chromosome loss) (Fenech et al. 2011), in comparison with point mutations that can accrue via a range of processes (e.g., base-pair substitutions, frame-shifts, deletions) (Lambert et al. 2005). Therefore, it is logical that a higher BMD would be observed for the MN endpoint than for the mutant endpoints, as we see here.

Although the aforementioned approach, which orders PoD across a series of sequential endpoints, is mechanistically appealing, we contend that it is inappropriate to directly compare BMDs across endpoints with different dynamic ranges of inducible responses (Fig. 5b). As specified in Slob (2017) endpoints with very large inducible ranges (i.e., many fold changes between control response and maximum inducible response) can be expected to yield very low BMD values as it requires very little effort to move the response above background to the BMR. This is especially apparent when comparing an endpoint with a relatively high theoretical maximum and very low detection limit (e.g., DNA adducts) to an endpoint such as % MN. For the MN endpoint, the maximum response generally observed is ten- to 20-fold above background (Fig. 5b); in contrast, the observed maximum responses for the DNA adduct and *lacZ* mutant endpoints are 100- to 1000-fold above background. This difference alone, regardless of the endpoints’ sequential involvement in the determination of the adverse outcome, will contribute to a BMD series, for a set BMR, whereby adduct frequency is lowest, followed by mutation and chromosomal damage. The problem is further illustrated by consideration of the cancer endpoint, which is highly constrained with respect to maximum response, as the data are quantal (i.e., animals are tumor bearing or not). Therefore, at a BMR of 100, 100% of the animals are tumor bearing, and thus it is not possible to have an effect size > 100%. However, for continuous endpoints, a BMR of 100 simply reflects a two-fold doubling of control, which is, in comparison, a small, and relatively easily achieved effect size (Sander et al. 2005). A 10% increase in the incidence of cancer may be of concern, but, in contrast, a 10% increase in adduct frequency may not constitute a significant disease-related change given the relative ease with which the latter endpoint can be moved away from the background. The approach presented by Slob (2017) to correct for cross-endpoint differences in theoretical maximum, will be applied to this and other genetic toxicity datasets in a forthcoming manuscript.

Recent works by Soeteman-Hernández et al. employed the BMD approach to examine empirical relationships between several genetic toxicity and carcinogenicity endpoints (Hernández et al. 2011; Soeteman-Hernandez et al. 2015, 2016). However, there is a paucity of data examining quantitative relationships between DNA damage sensitivity (i.e., adduct sensitivity) and mutagenic sensitivity. In our study, when examining the relationship between DNA adducts and *lacZ* mutants, we were only able to see a cross-tissue relationship when the comparison was based on the BMD metric. This is the first demonstration of an empirical relationship between DNA adduct induction and mutation induction across several tissues. For BaP, the results suggest a consistent, proportional conversion of adducts to mutations across tissues. DNA adduct frequency is a biomarker of exposure, whereas mutations and chromosomal damage are biomarkers of effect and, therefore, DNA adduct data are currently used in more of a qualitative manner to establish exposure and genotoxic potential of a compound (Sander et al. 2005). Additional research will be required to demonstrate whether proportionality between DNA adducts and mutations exist for other chemicals, but if this is confirmed, it may enable us to reliably use DNA adduct data in a more quantitative manner. This would be advantageous since DNA adduct frequency is much more readily assessed across tissues, and does not require the use of transgenic rodents.

Finally, it is important to note that this novel analysis approach need not be restricted to newly generated data such as those presented here. Analysis of published data, such as that previously presented by our group (Wills et al. 2016a, b), can permit the determination of potency or sensitivity rankings across compounds, tissues, cell types, and/or exposure regimens. The results of such analyses can reduce the necessity of additional studies, thus contributing to an overall reduction in animal use without sacrificing the precision of metrics used for human health risk assessment.

## Conclusions

We documented BaP-induced genetic damage across all endpoints and tissues examined, even at very low doses (e.g., 0.2 mg/kg BW/day for spleen adducts). By ranking BMD CIs across tissues for a given endpoint, we observed that sensitivity varied significantly across tissues in regards to induced *lacZ* MF, and the differences were more pronounced in comparison with the DNA adduct endpoint. We were unable to observe cell population-specific sensitivity differences for *Pig*-*a* MF and MN frequency endpoints. We demonstrated that the cross-tissue BMD trend was similar for both DNA adducts and *lacZ* mutants, and that this trend is consistent with tissue-specific differences in metabolism, proliferation, and repair. Moreover, we showed quantitative evidence that cross-tissue BMD_100_s for DNA adduct induction are proportional to BMD_100_s for *lacZ* mutant induction, illustrating that the tissue-specific conversion of adducts to mutations is likely consistent, and that this empirical dependency is consistent across the seven tissues examined. Finally, our cross-endpoint comparisons of BMD_100_ values raised questions regarding the validity of comparisons based on a fixed BMR value. Cross-endpoint comparisons of BMD metrics using a fixed BMR have become unfortunately common, but this work and the earlier work of Slob (2017) indicate that, for comparisons to be meaningful, BMR values must be scaled according to endpoint-specific theoretical maxima. Overall, the BMD-approach employed herein permitted robust comparisons of responses across tissues and endpoints, and the information obtained adds valuable information to our mechanistic understanding of how BaP induces an array of genetic damage across several tissues. BMD rankings within an endpoint, and empirical comparisons across endpoints, contribute to an improved understanding of tissue-specific, chemically induced genetic damage, and this knowledge can provide a foundation for the selection of tissues, endpoints and BMRs for use in human health risk assessments. To enhance current understanding regarding the tissue-specific fixation of chemically induced genetic damage, future work should continue to examine the cross-tissue relationships between chemically induced mutations and/or chromosomal damage and induced DNA damage.

## Electronic supplementary material

Below is the link to the electronic supplementary material.

Supplementary material 1 (DOCX 1081 kb)
