# Supplementary material for: Benchmark dose analyses of multiple genetic toxicity endpoints permit robust, cross-tissue comparisons of MutaMouse responses to orally delivered benzo[a]pyrene
Source: Arch Toxicol. 2017 Nov 24;92(2):967–82. doi: 10.1007/s00204-017-2099-2 (PMC5818629; doi:10.1007/s00204-017-2099-2)
Supplement: Supplementary file 1 — Supplementary material 1 (DOCX 1081 kb) [file 204_2017_2099_MOESM1_ESM.docx]

SUPPLEMENTAL MATERIAL FOR:

**Benchmark Dose Analyses of Multiple Genetic Toxicity Endpoints Permit Robust, Cross-Tissue Comparisons of MutaMouse Responses to Orally Delivered Benzo[*a*]pyrene**

Alexandra S. Long, John W. Wills, Dorothy Krolak, Matthew Guo, Stephen D. Dertinger, Volker M. Arlt^Ŧ^, Paul A. White^Ŧ^

^Ŧ^ Contributed Equally

*Table of Contents:*

Supplemental methods.................pg. 2

Supplemental Table.................... pg. 3

Supplemental Figure Captions... pg. 4

Supplemental Figure 1............... pg. 6

Supplemental Figure 2............... pg. 9

Supplemental Figure 3............... pg. 10

Supplemental Figure 4............... pg. 11

SUPPLEMENTARY METHODS:

The lung and heart were excised attached, and the heart and right lobe of the lung were clamped off and removed. The left lung lobe was then perfused with 10% neutral buffered formalin (Surgipath, Winnipeg, MB) via the trachea until inflated. The duodenum was excised, flushed with phosphate buffered saline, followed by 10% NBF. The caudate lobe of the liver was excised and all tissues were immediately preserved in 10% NBF for immunohistochemical analysis. Formalin-fixed tissue samples were embedded in paraffin the day following necropsy and the resulting formalin-fixed paraffin-embedded tissues were sectioned to 4-μm-wide slices. Tissue slices were mounted on slides and dried overnight. The slides were analysed for cell proliferation (ki67) using ki67 antibody (clone TEC-3) (DAKO, Burlington, ON). All slides were visualised on a Leica DM 4500 B microscope equipped with an Olympus DP 70 camera. Image acquisition and data analysis were conducted using Visiopharm Integrator System (v.3.2.8.0, Hoersholm, Denmark). Ki67 index (i.e., proliferation index) was calculated as the frequency of ki67-positive stained cells.

SUPPLEMENTARY TABLES

**Supp. Table I.** BMD_100_ values and two-sided 90% confidence intervals (i.e., the BMDL & BMDU) for each endpoint and tissue/cell population. BMDs were determined (in mg BaP/kg BW/day) using the 4-parameter exponential model in PROAST. BM = Bone marrow; Lv = Liver; GS = Glandular stomach; SI = Small intestine; Lg = Lung; Sp = Spleen; Kd = Kidney, RET = reticulocyte; RBC = red blood cell; NCE = normochromatic erythrocyte.

| **Tissue** | BMD_100_ | BMDL | BMDU | BMDU-BMDL Ratio |
| --- | --- | --- | --- | --- |
| **Adducts** | | | | |
| **SI** | 0.0278 | 0.00255 | 0.0722 | 28.3 |
| **BM** | 0.0286 | 0.0196 | 0.0661 | 3.37 |
| **Sp** | 0.0728 | 0.0409 | 0.118 | 2.89 |
| **GS** | 0.106 | 0.0544 | 0.200 | 3.68 |
| **Lg** | 0.110 | 0.0873 | 0.136 | 1.56 |
| **Bd** | 0.123 | 0.0792 | 0.190 | 2.40 |
| **Kd** | 0.140 | 0.0979 | 0.193 | 1.97 |
| **Lv** | 0.158 | 0.122 | 0.199 | 1.63 |
| ***lacZ*** | | | | |
| **SI** | 0.878 | 0.738 | 1.03 | 1.39 |
| **Sp** | 1.904 | 1.34 | 2.55 | 1.90 |
| **BM** | 2.25 | 1.81 | 2.75 | 1.52 |
| **GS** | 5.55 | 4.20 | 7.06 | 1.68 |
| **Lg** | 7.14 | 5.96 | 8.42 | 1.41 |
| **Lv** | 11.7 | 8.47 | 14.6 | 1.73 |
| **Kd** | 12.1 | 8.34 | 16.3 | 1.96 |
| **MN** | | | | |
| **NCEs** | 10.2 | 8.35 | 12.2 | 1.47 |
| **RETs** | 10.3 | 7.75 | 13.1 | 1.69 |
| ***Pig-a*** | | | | |
| **RBCs** | 4.47 | 2.78 | 6.46 | 2.33 |
| **RETs** | 5.71 | 3.95 | 9.37 | 2.37 |

SUPPLEMENTARY FIGURE CAPTIONS

**Supp. Figure 1.** PROAST model fits for **(a)** DNA adducts, **(b)** *lacZ* mutations, **(c)** *Pig-a* mutations, and **(d)** micronucleus frequency data. Small points are individual observations, larger circles are geometric means. The curves reflect the fitted four-parameter exponential model. Horizontal and vertical dashed lines represent BMR of 100% and BMD_100_, respectively. The BMD_100_, BMDL, and BMDU are inset. Note that the control group (i.e., dose zero) is situated at minus infinity on a log_10_-scale and a ‘placeholder’ on the X-axis is required to permit visualization. The horizontal dotted line represents the response limit of quantification used in the statistical calculations.

**Supp. Figure 2.** Autoradiographic profiles of DNA adducts, measured by thin-layer chromatography ^32^P-postlabelling, in various tissues exposed to 50 mg/kg BW/day BaP. The adduct profiles shown are representative of the cross-tissue pattern observed throughout the dose range examined. No BaP-DNA adducts were detected in control tissues. Solvent conditions for the separation of BaP-derived DNA adducts were as follows: D1, 1.0 M sodium phosphate, pH 6.0; D3, 3.5 M lithium-formate, 8.5 M urea, pH 3.5; D4, 0.8 M lithium chloride, 0.5 M Tris, 8.5 M urea, pH 8.0. The origins, at the bottom left-hand corners, were cut off before imaging. The arrow indicates the adduct spot of 10-(deoxyguanosin-*N*^2^-yl)-7,8,9-trihydroxy-7,8,9,10-tetrahydro-BaP (dG-*N*^2^-BPDE) used for quantitation.

**Supp. Figure 3.** Benzo[*a*]pyrene dose-response data (± standard error) for DNA adducts with the results plotted on a restricted linear y-axis in order to visualize responses at low doses that were obscured when plotted on a log_10_ y-axis (as in **Figure 1a**). Statistical results for the overall dose–response relationship are presented for each tissue. The level of significance for the custom contrast results for each dose vs. control are indicated as follows: a = p<0.0001; b = p<0.001; c = p<0.01; d = p<0.05. BM = Bone marrow; Lv = Liver; GS = Glandular stomach; SI = Small intestine; Lg = Lung; Sp = Spleen; Kd = Kidney; Bd = Bladder.

**Supp. Figure 4.** BMD_100_ ratio of DNA adducts to *lacZ* mutations for liver (Lv), lung (Lg), and small intestine (SI) versus the Ki-67 index. Ki-67 index was determined via comparative immunohistochemical analyses of FFPE tissues slices from control and exposed animals.

SUPPLEMENTARY FIGURES


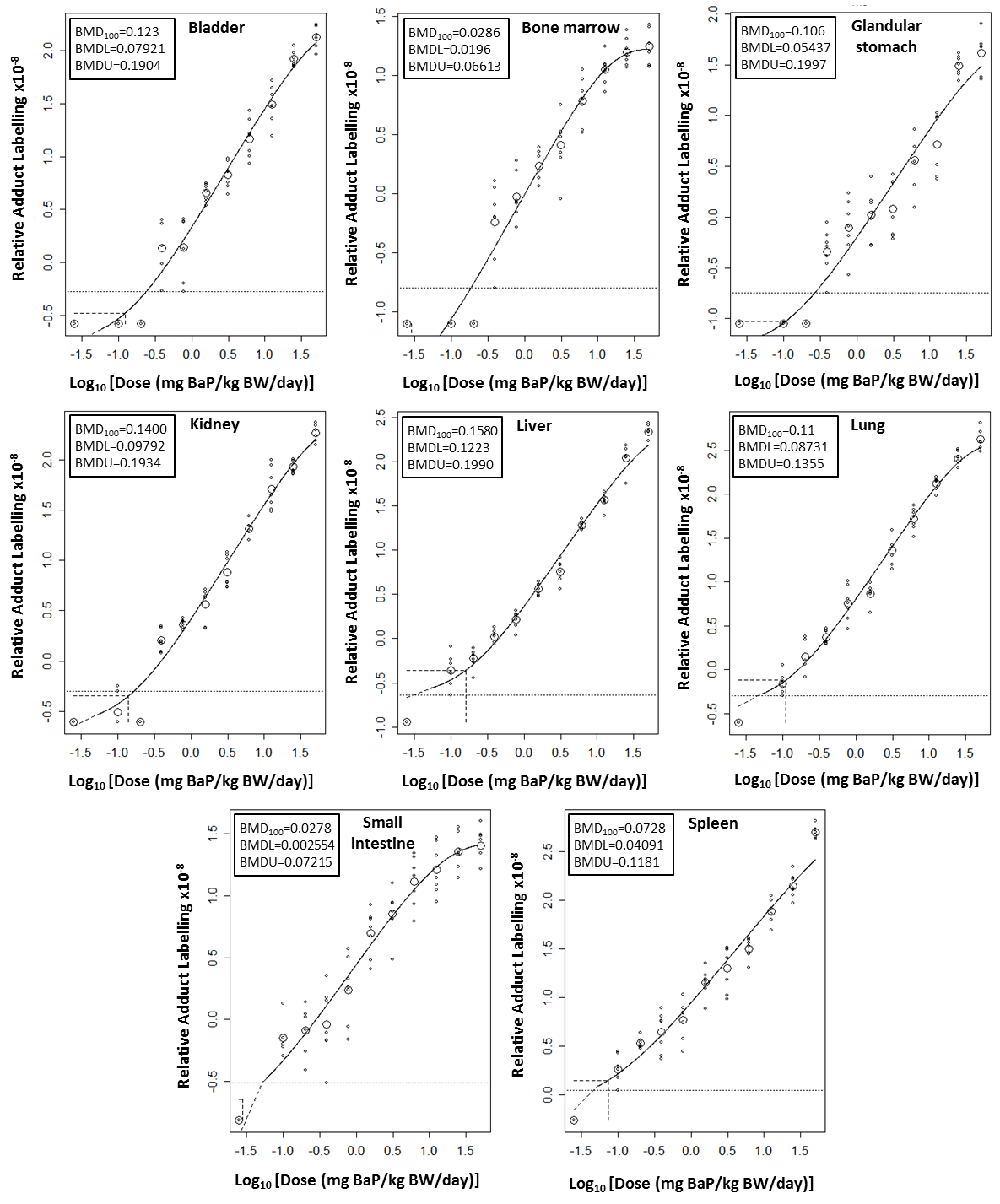


**Supp. Figure 1a.**


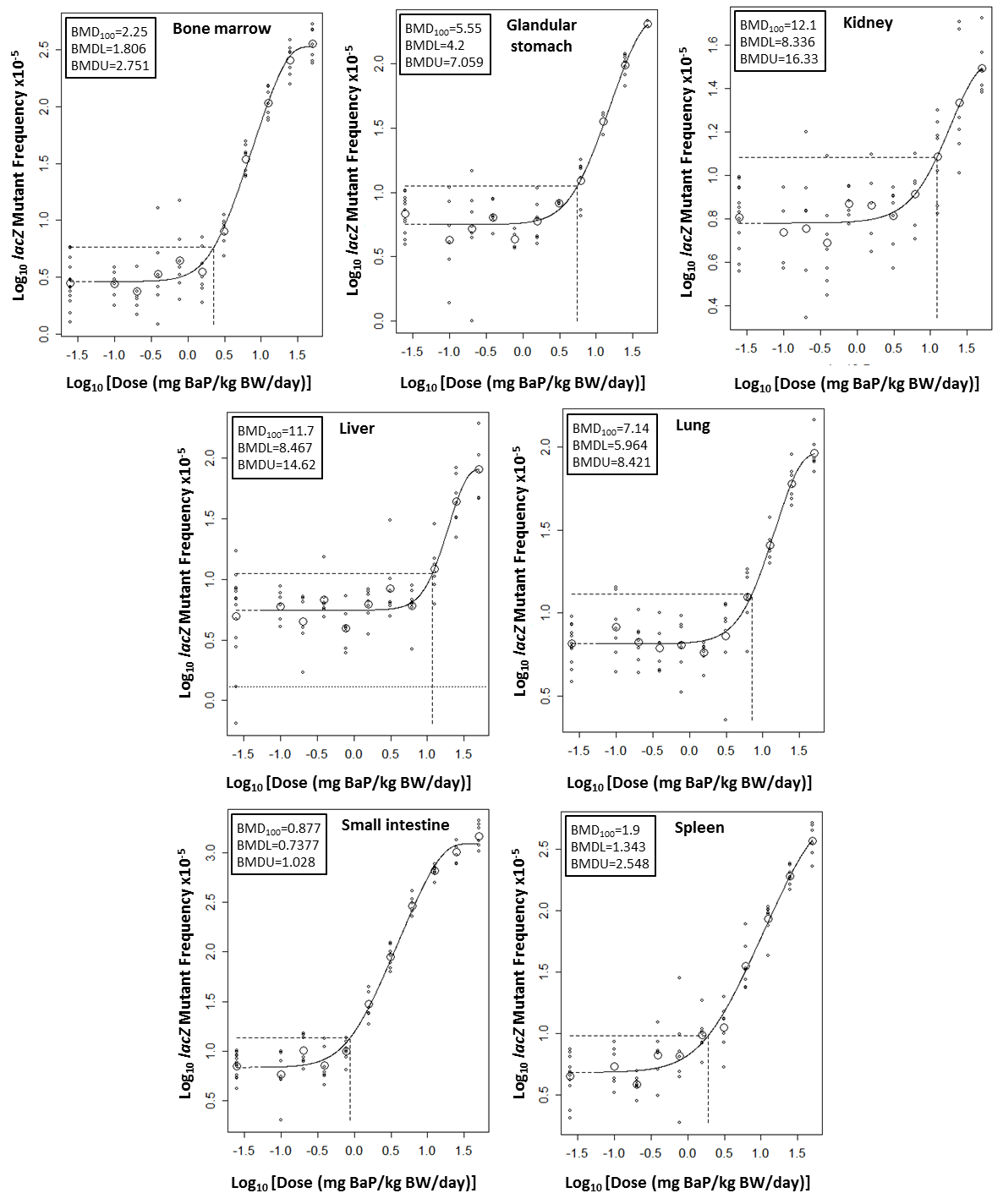


**Supp Figure 1b.**


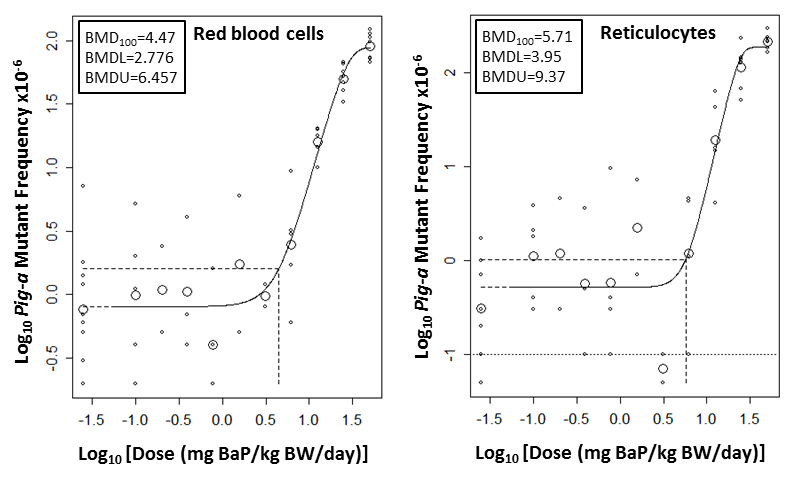


**Supp. Figure 1c.**


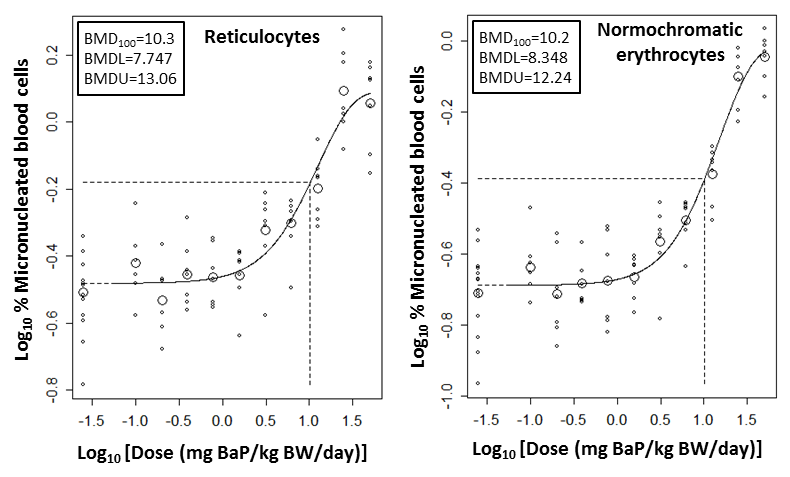


**Supp. Figure 1d.**

**
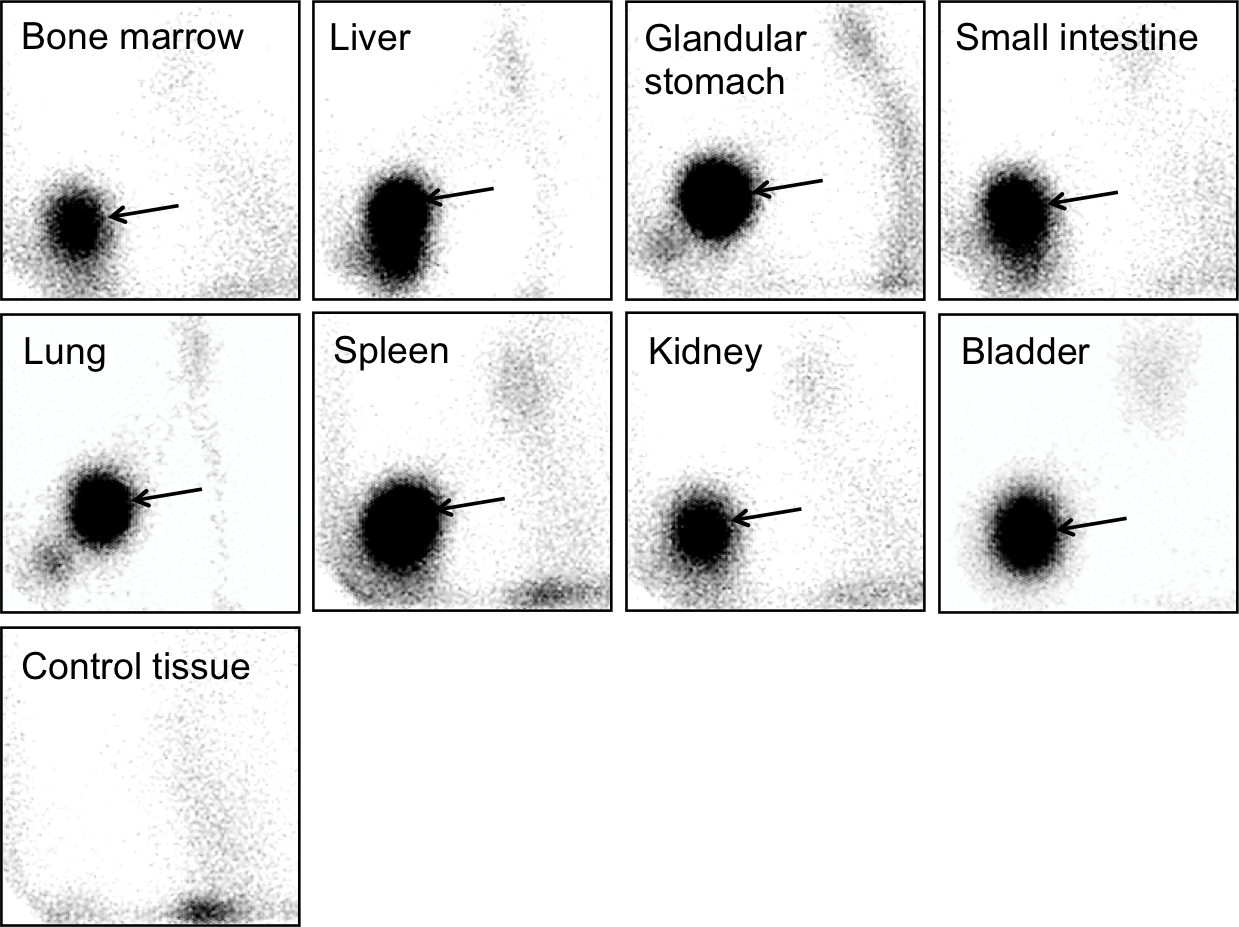
**

**Supp. Figure 2.**

**
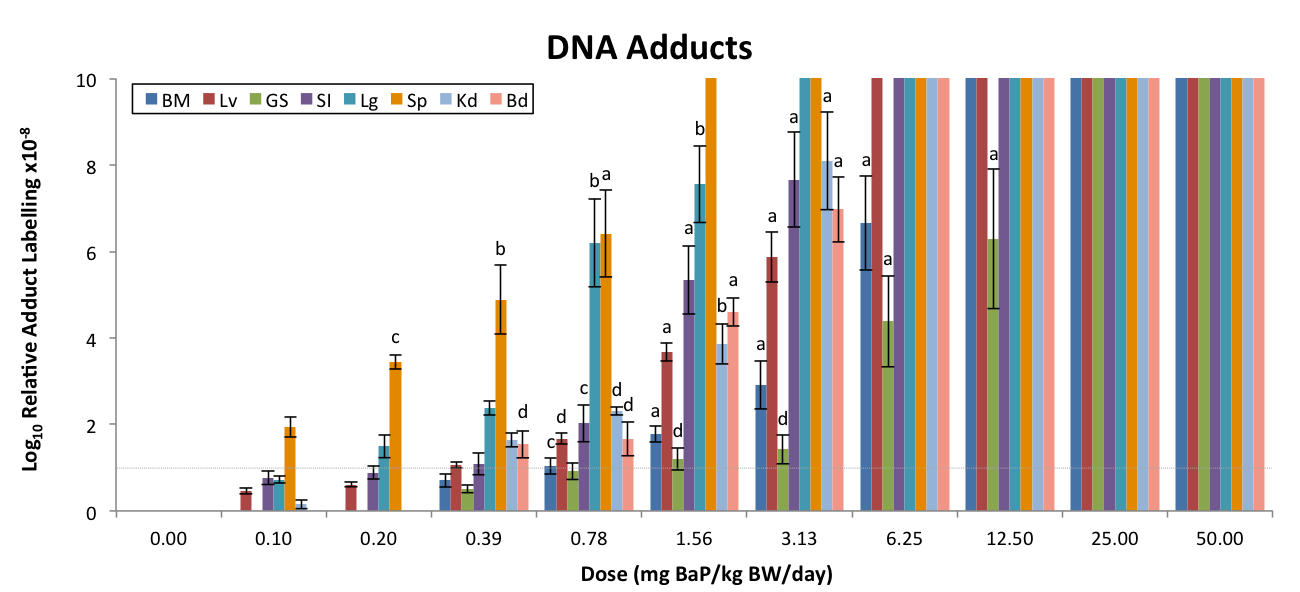
Supp. Figure 3.**


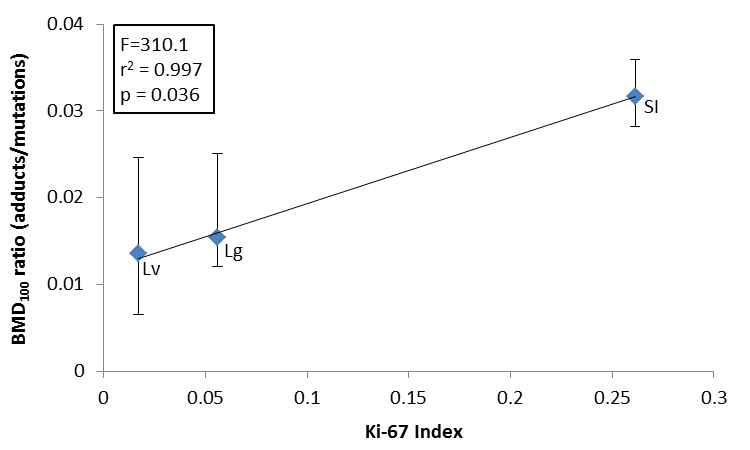


**Supp. Figure 4.**
